# Survival changes in patients with small cell lung cancer and disparities between different sexes, socioeconomic statuses and ages

**DOI:** 10.1038/s41598-017-01571-0

**Published:** 2017-05-02

**Authors:** Shuncong Wang, Jianjun Tang, Tiantian Sun, Xiaobin Zheng, Jie Li, Hongliu Sun, Xiuling Zhou, Cuiling Zhou, Hongyu Zhang, Zhibin Cheng, Haiqing Ma, Huanhuan Sun

**Affiliations:** 1 Department of Oncology, The Fifth Affiliated Hospital of Sun Yat-sen University, Zhuhai, Guangdong 519000 China; 2 Department of Gastroenterology, Cancer Hospital of Jiangxi Province, Nanchang, Jiangxi 330029 China; 3 Department of Hematology, The First Affiliated Hospital of Sun Yat-sen University, Guangzhou, Guangdong 510080 China; 4 Department of Respiration, The Fifth Affiliated Hospital of Sun Yat-sen University, Zhuhai, Guangdong 519000 China; 5 Department of Breast and Thyroid Surgery, The First Affiliated Hospital of Sun Yat-sen University, Guangzhou, Guangdong 510080 China; 6 Department of Pathology, University of Michigan, Ann Arbor, MI 48201 USA

## Abstract

Small cell lung cancer (SCLC), as a proportion, makes up only 15–17% of lung cancer cases. The development of treatments for SCLC has remained stagnant for decades, and SCLC is expected to persist as a threat to human health. To date, no publications based on large populations have been reported. We calculated survival changes in patients with SCLC during each decade between 1983 and 2012 to determine the roles of race, sex, age, and socioeconomic status (SES) on survival rates based on the Surveillance, Epidemiology, and End Results (SEER) registries. In total, 106,296 patients with SCLC were identified, with the overall incidence per 100,000 decreasing each decade from 9.6 to 7.8 to 5.8. The median survival for SCLC remained 7 months, and the 12-month relative survival rates (RSRs) remained relatively stable at 32.9%, 33.2% and 33.2% during each decade. The 5-year RSRs significantly improved from 4.9% to 5.9% to 6.4% during each decade, but remained extremely low. In addition, a narrowing of the survival gaps among SES groups and stable survival gaps between sexes were observed. Although the incidence of SCLC decreased during each decade, the overall survival remained relatively stable, highlighting the urgency of developing novel treatments and the importance of prevention and early detection.

## Introduction

Lung cancer is estimated to be the second most common cancer type and the leading cause of cancer-related death in both sexes, with an estimated 116,990 cases in males and 105,510 in females^1^. Small-cell lung cancer (SCLC), accounting for approximately 15% to 17% of all diagnosed lung cancers, is characterized by a high invasiveness, short doubling time, high growth fraction and ease of metastasis upon diagnosis^2, 3^. Classified as a neuroendocrine tumor despite its differences with non-small cell lung cancer (NSCLC), SCLC is distinctly different from extrapulmonary small cell carcinoma in disease progression, prognosis and etiology^4^.

Radiotherapy plus platinum-based chemotherapy has been the standard treatment for most SCLC cases for 30 years and has reached an efficiency plateau. Surgery is only an option for a small proportion of limited stage patients based on Veterans Administration Lung Study Group (VALSG) staging^5–8^. In addition and in sharp contrast to NSCLC, for which survival has improved remarkably in the past few decades due to the development of EGFR, ROS1, and ALK inhibitors among others, no major developments have been made in SCLC treatment, including targeted agents, such as EGFR-TKI, BCR-ABL TKIs, or mTOR inhibitors^9–11^. These factors have limited improvements of the currently dismal survival rates for SCLC. In addition, the average cost for SCLC treatment remains enormous, at 11,556 pounds, or 15,418 Euros, for limited disease and 12,482 Euros for extensive disease^12, 13^. More radical chemotherapy or maintenance of chemotherapy did not significantly improve patients’ survival^8, 14–17^. These trends have challenged healthcare legislators as the current treatment plateau has not been overcome by additional financial expenditures, especially given the already high financial burden of SCLC. In addition to the lack of treatment effectiveness, approximately 70% of SCLC diagnoses are already at the point of extensive disease, indicating that more emphasis should be placed on clinical awareness of screening and early detection^18^. Intuitively, SCLC survival is expected to remain constant, but no relevant large population-based publications have been reported. Previous publications on SCLC survival rates focused on specific patient populations at certain stages or patients receiving chemotherapy, prophylactic cranial irradiation and surgery^19–23^. This study, using period analyses, aimed to evaluate the survival changes in each decade from 1983 to 2012 on the basis of the Surveillance, Epidemiology, and End Results (SEER) database.

Additionally, racial and socioeconomic status (SES) disparities in the U.S. health care system are of great concern and have been shown to influence survival in many malignancies^24, 25^. Preliminary data have shown that SES can also affect survival for extensive stages of SCLC, indicating the need for amelioration in healthcare policies to balance this survival disparity^26^. This study determined the survival changes over three decades and clarified the roles of sex, age, race and SES on the survival of patients with SCLC based on the SEER database.

## Results

### Incidence of SCLC over three decades

**Figure 1:**
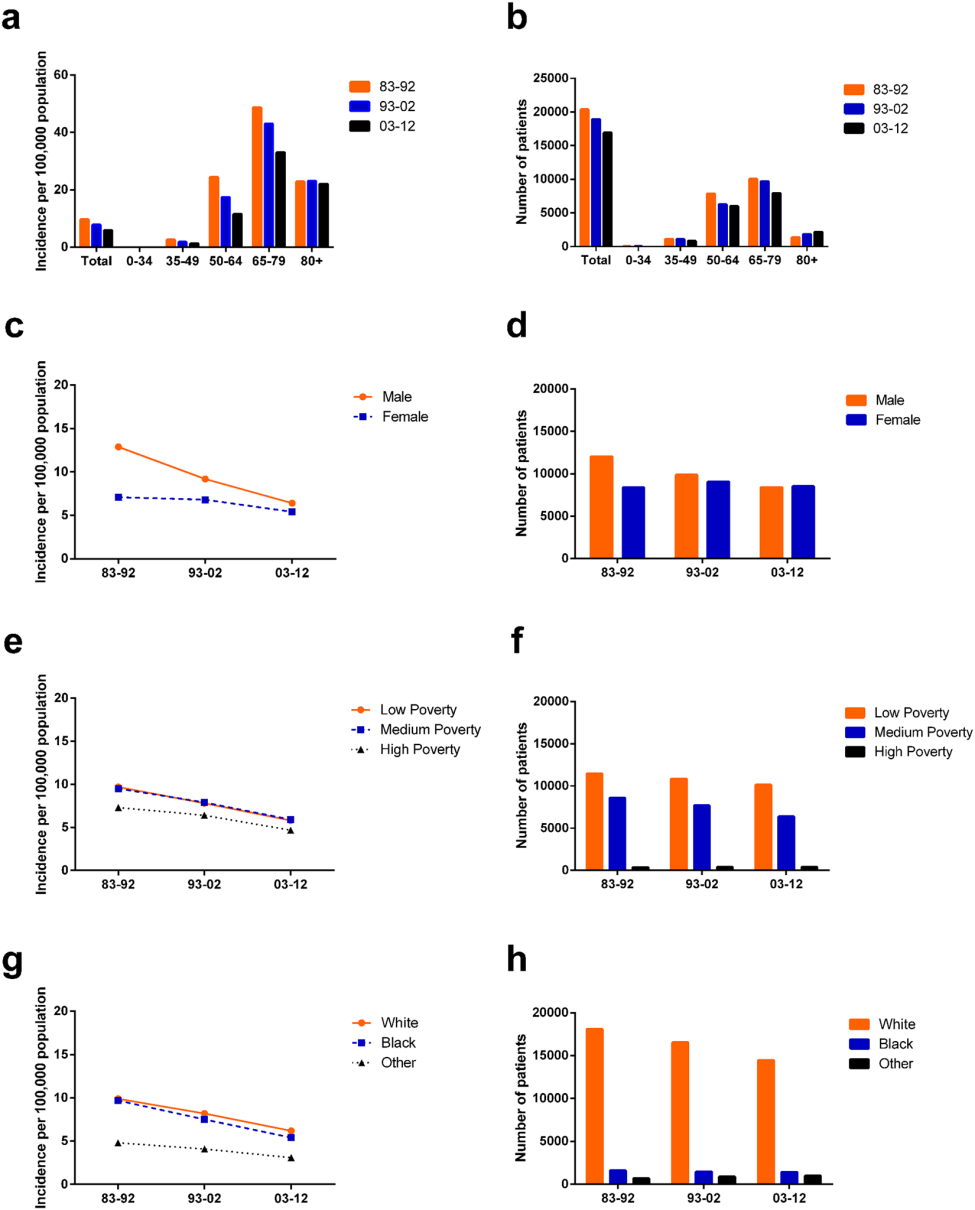
Summary incidences of patients diagnosed as having SCLC between 1983 and 2012 at the original nine SEER sites. Incidence (**a**) and number (**b**) of SCLC cases are shown by age group (total and ages 0–34, 35–49, 50–64, 65–79, and 80+ years) and calendar period. Incidence and number of SCLC cases are grouped by sex (**c**,**d**), SES (**e**,**f**), and race (**g**,**h**), respectively.

To guarantee the comparability of incidence data over three decades by extracting data from the same registry sites, we extracted incidence data from the nine original registry sites in the SEER database. A total of 56,220 cases diagnosed with SCLC between 1983 and 2012 were identified. The overall incidence of SCLC per 100,000 decreased each decade over time to 9.6, 7.8 and 5.8, as illustrated in Fig. 1 and Supplementary Table S1. This trend of reduction in incidence can be seen in most age groups, with large decreases in some groups: from 24.4 to 17.4 to 11.5 in the 50–64 age group and from 48.7 to 43.0 to 33.1 in the 65–79 age group (Fig. 1a, Suppl. Table S1). However, incidence remained stable in the 0–34 and older than 80 age groups. Incidence was highest in the 65–79 age group in all three decades. The SCLC patient number decreased in most groups except for the over 80 group, in which the patient number kept increasing over time (Fig. 1b, Suppl. Table S1).

Incidence differences were observed between sexes, with a higher incidence per 100,000 in males (12.9 vs. 7.1 in 1983–1992, 9.2 vs. 6.8 in 1993–2002, and 6.4 vs. 5.4 in 2003–2012; Fig. 1c, Suppl. Table S1). More importantly, the incidence gaps between sexes kept shrinking due to the rapidly declining incidence in males and stable incidence in females. The patient number in both sexes became similar over the last two decades due to decreasing male cases and a stable number of female cases (Fig. 1d).

### SCLC incidence by SES and by race

As illustrated in Fig. 1e,f and Supplementary Table S1, ever-decreasing incidence rates over the three decades were observed in all SES groups. The medium-poverty group (from 9.5 to 7.9 to 5.9) shared a similar incidence with the low-poverty group (from 9.7 to 7.8 to 5.8) each decade and had a higher incidence than the high-poverty group (from 7.3 to 6.4 to 4.7). The number of SCLC patients continuously decreased in the low-poverty and medium-poverty groups, whereas in the high-poverty group, it remained relatively stable (Fig. 1f).

The incidence per 100,000 patients in all racial groups decreased over the three decades, with Whites showing a higher SCLC incidence than Blacks or Others. The incidence gaps between Whites and Others kept narrowing each decade (from 5.1 to 4.1 to 3.1), whereas the incidence gaps between Whites and Blacks widened slightly each decade (Fig. 1g, Suppl. Table S1.) The number of White and Black SCLC patients decreased each decade; however, the number of patients in the Others group increased slightly (Fig. 1h).

### Survival for SCLC patients over three decades

A total of 106,439 patients with SCLC between 1983 and 2012 at 18 registry sites were identified, with the five-year survival rate improving from 4.9% to 5.9% to 6.4% each decade (*p* < 0.0001). However, the median survival remained 7 months in each decade.

**Figure 2:**
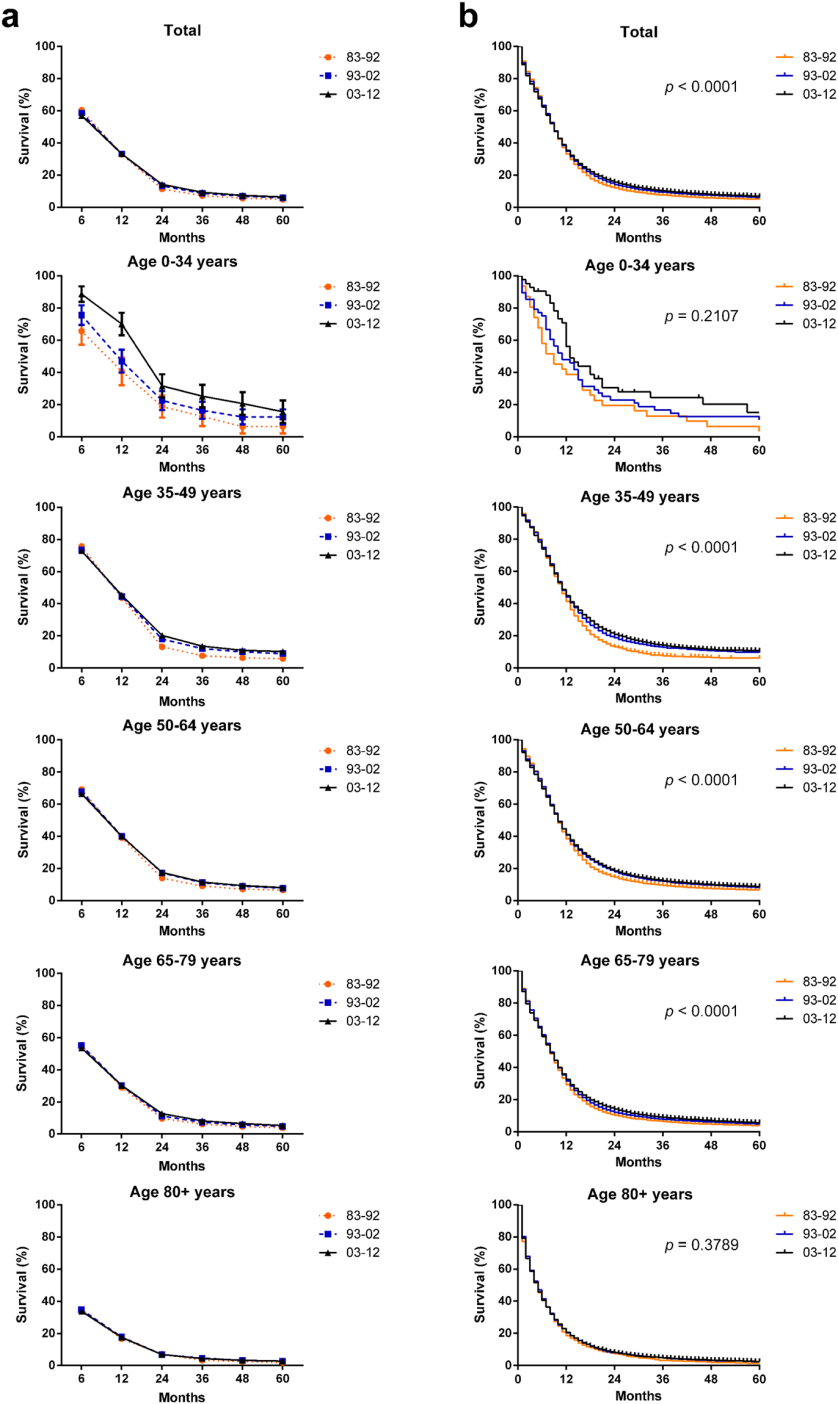
Trends in five-year relative survival rates (**a**) and Kaplan-Meier survival analyses (**b**) for patients with SCLC at 18 SEER sites between 1983 and 2012 according to age group (total and ages 0–34, 35–49, 50–64, 65–79, and 80+ years) and calendar period.

Relative survival rates (RSRs) in patients with SCLC remained relatively stable in the total population and in almost all age groups over the three decades (Table 1 and Fig. 2a). The 12-month RSRs remained relatively stable in the total population and all age groups across the three decades. However, a slight survival improvement could be seen in the 36-month RSRs, improving from 7.2% to 8.6% to 9.3% over the three decades, with a larger increase in the first two decades (*p* < 0.0001). More importantly, the survival improvement over the three decades was age-dependent, with a greater survival improvement in younger patient groups. The improvement in RSR over the three decades was followed up for 5 years. Significantly improved survival times over the three decades in the total population and in the 35–49, 50–64 and 65–79 age groups were also confirmed by Kaplan-Meier curves (*p* < 0.0001, Fig. 2b). Furthermore, the trends toward increased long-term survival and age-dependent survival improvement were also seen in the Kaplan-Meier curves.

**Table 1:** Relative survival rates of SCLC patients during the periods of 1983–1992, 1993–2002, and 2003–2012 at 18 SEER sites.

Age Group	Decade		
	1983–1992	1993–2002	2003–2012
6-Mo RS			
Total	60.4 ± 0.3 (20902)	58.6 ± 0.3 (33618)***	57.0 ± 0.2 (50608)***
0–34	65.7 ± 8.4 (32)	75.6 ± 6.1 (49)	88.7 ± 4.8 (44)
35–49	75.7 ± 1.3 (1134)	73.5 ± 1.0 (1907)	72.9 ± 0.9 (2652)
50–64	69.2 ± 0.5 (8024)	67.9 ± 0.4 (11300)	66.5 ± 0.4 (18229)
65–79	55.4 ± 0.5 (10311)	55.2 ± 0.4 (17059)	53.5 ± 0.3 (23900)**
80+	34.6 ± 1.3 (1401)	34.8 ± 0.9 (3303)	33.8 ± 0.6 (5783)
12-Mo RS			
Total	32.9 ± 0.3	33.2 ± 0.3	33.2 ± 0.2
0–34	40.7 ± 8.7	47.0 ± 7.1	70.1 ± 7.0
35–49	43.7 ± 1.5	44.6 ± 1.1	45.4 ± 1.0
50–64	39.1 ± 0.6	40.2 ± 0.5	40.0 ± 0.4
65–79	29.0 ± 0.5	30.2 ± 0.4	30.3 ± 0.3
80+	16.7 ± 1.1	17.8 ± 0.7	17.5 ± 0.5
24-Mo RS			
Total	11.4 ± 0.2	13.3 ± 0.2***	14.2 ± 0.2*
0–34	18.8 ± 6.9	22.5 ± 6.0	31.6 ± 7.3
35–49	13.2 ± 1.0	18.0 ± 0.9**	20.3 ± 0.8
50–64	14.0 ± 0.4	17.3 ± 0.4***	17.5 ± 0.3
65–79	9.7 ± 0.3	11.3 ± 0.3**	12.7 ± 0.2***
80+	7.0 ± 0.8	6.9 ± 0.5	6.9 ± 0.4
36-Mo RS			
Total	7.2 ± 0.2	8.6 ± 0.2***	9.3 ± 0.1**
0–34	12.6 ± 5.9	16.4 ± 5.3	25.2 ± 7.1
35–49	7.7 ± 0.8	12.1 ± 0.8**	13.6 ± 0.7
50–64	9.1 ± 0.3	11.2 ± 0.3***	11.5 ± 0.3
65–79	6.2 ± 0.3	7.3 ± 0.2*	8.2 ± 0.2*
80+	3.3 ± 0.6	4.6 ± 0.4	4.4 ± 0.3
60-Mo RS			
Total	4.9 ± 0.2	5.9 ± 0.1***	6.4 ± 0.1**
0–34	6.3 ± 4.3	12.3 ± 4.7	15.5 ± 7.0
35–49	5.9 ± 0.7	9.0 ± 0.7*	10.3 ± 0.6
50–64	6.5 ± 0.3	7.8 ± 0.3*	8.1 ± 0.2
65–79	3.9 ± 0.2	4.8 ± 0.2*	5.3 ± 0.2
80+	1.8 ± 0.5	2.8 ± 0.4	2.8 ± 0.3

Data are mean ± standard error of the mean, with number of patients in parentheses. Abbreviations: Mo, month; RS, relative survival; SEM, standard error of the mean. *p < 0.01, **p < 0.001, and ***p < 0.0001 for comparisons with the preceding decade.

**Figure 3:**
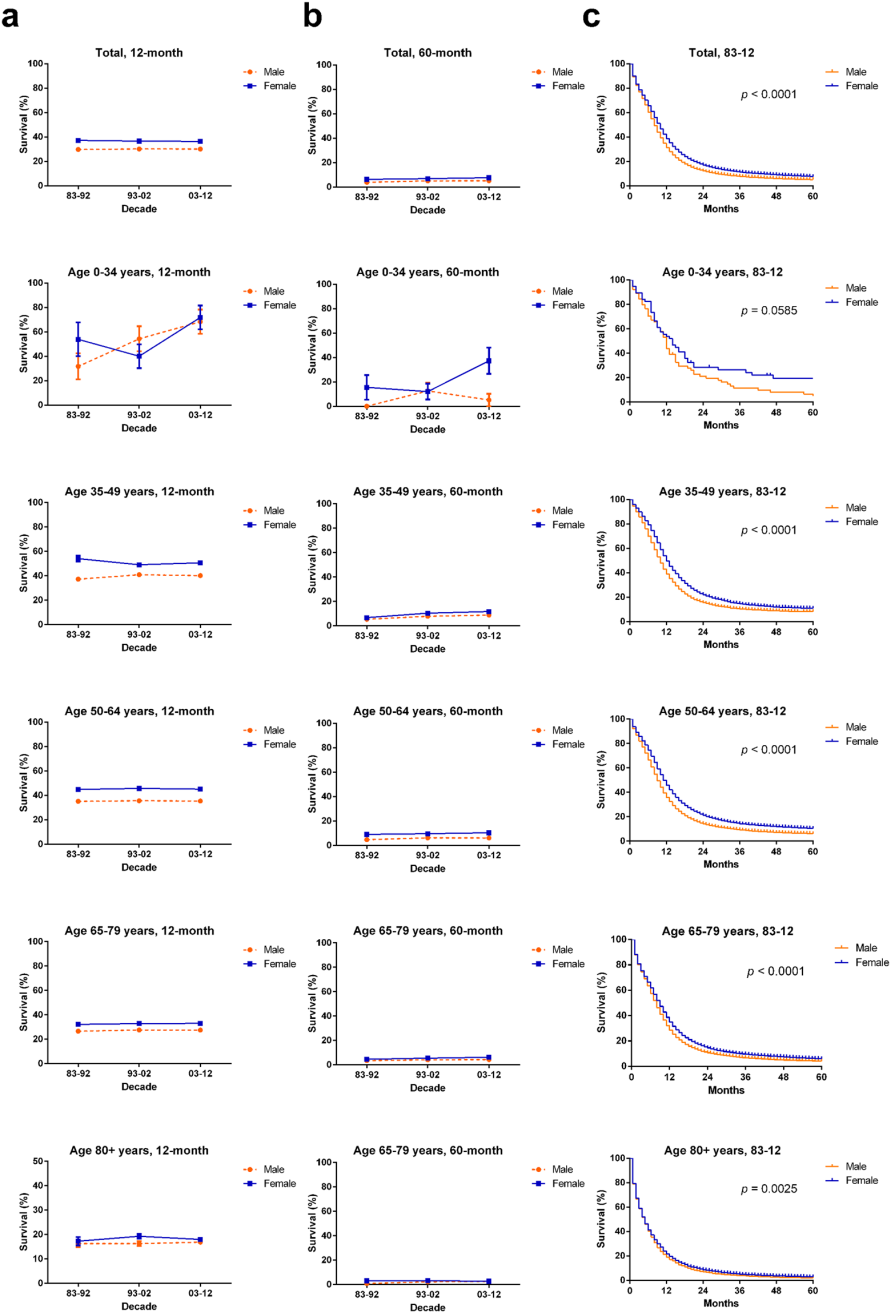
12-month (**a**), 60-month (**b**) relative survival rates and Kaplan-Meier survival analyses from 1983 to 2012 (**c**) for patients with SCLC at 18 SEER sites according to sex by age group (total and ages 0–34, 35–49, 50–64, 65–79, and 80+ years).

As illustrated in Table 2 and Fig. 3, the 12-month RSRs in both sexes remained relatively stable over the three decades. However, slight improvements could be seen for both sexes in the 60-month RSRs, as well as in the 24- and 36-month RSRs (Table 2, Fig. 3, Suppl. Figure S1, Suppl. Table S2). A survival advantage in the 12-month RSRs was determined in females (37.2% vs. 29.8%, *p* < 0.0001), and the survival gap between sexes remained relatively stable each decade (36.6% vs. 30.2% in 1993–2002 and 36.4% vs. 30.1% in 2003–2012, *p* < 0.0001; Table 2, Fig. 3a). The survival advantage in females and stable survival gap could also be seen in the Kaplan-Meier survival analyses and 60-month RSRs, as well as in the 24- and 36-month RSRs (*p* < 0.0001) (Fig. 3b, Suppl. Table S2, Suppl. Figure S2 and Suppl. Figure S3).

**Table 2:** 12-month 60-month relative survival rates of SCLC patients according to sex, age group, and calendar period from 1983 to 2012 at 18 SEER sites.

Decade	Age Group	Sex	
		Male	Female
83–92	12-Mo RS		
	Total	29.8 ± 0.4 (12341)	37.2 ± 0.5 (8561)***
	0–34	31.7 ± 10.7 (19)	53.9 ± 13.8 (13)
	35–49	37.2 ± 1.8 (698)	54.0 ± 2.4 (436)***
	50–64	35.1 ± 0.7 (4759)	44.9 ± 0.9 (3265)***
	65–79	26.6 ± 0.6 (6026)	32.2 ± 0.7 (4285)***
	80+	16.3 ± 1.4 (839)	17.3 ± 1.7 (562)
	60-Mo RS		
	Total	3.9 ± 0.2 (12341)	6.3 ± 0.3 (8561)***
	0–34	0.0 ± 0.0 (19)	15.5 ± 10.1 (13)
	35–49	5.4 ± 0.9 (698)	6.7 ± 1.2 (436)
	50–64	4.7 ± 0.3 (4759)	9.0 ± 0.5 (3265)***
	65–79	3.5 ± 0.3 (6026)	4.6 ± 0.3 (4285)
	80+	0.9 ± 0.4 (839)	3.1 ± 0.9 (562)
93–02	12-Mo RS		
	Total	30.2 ± 0.4 (17657)	36.6 ± 0.4 (15961)***
	0–34	54.3 ± 10.2 (24)	40.0 ± 9.8 (25)
	35–49	40.9 ± 1.5 (1045)	49 ± 1.7 (862)**
	50–64	35.6 ± 0.6 (6139)	45.7 ± 0.7 (5161)***
	65–79	27.6 ± 0.5 (8813)	32.9 ± 0.5 (8246)***
	80+	16.3 ± 1.0 (1636)	19.3 ± 1.0 (1667)
	60-Mo RS		
	Total	5.0 ± 0.2 (17657)	6.9 ± 0.2 (15961)***
	0–34	12.6 ± 6.8 (24)	12.0 ± 6.5 (25)
	35–49	7.8 ± 0.8 (1045)	10.4 ± 1.0 (862)
	50–64	6.2 ± 0.3 (6139)	9.6 ± 0.4 (5161)***
	65–79	4.2 ± 0.2 (8813)	5.5 ± 0.3 (8246)**
	80+	2.3 ± 0.5 (1636)	3.2 ± 0.5 (1667)
03–12	12-Mo RS		
	Total	30.1 ± 0.3 (25577)	36.4 ± 0.3 (25031)***
	0–34	68.3 ± 9.9 (22)	71.8 ± 9.8 (22)
	35–49	40.1 ± 1.4 (1307)	50.6 ± 1.4 (1345)***
	50–64	35.4 ± 0.5 (9607)	45.1 ± 0.5 (8622)***
	65–79	27.5 ± 0.4 (11881)	33.0 ± 0.4 (12019)***
	80+	16.9 ± 0.8 (2760)	18.0 ± 0.7 (3023)
	60-Mo RS		
	Total	5.1 ± 0.2 (25577)	7.6 ± 0.2 (25031)***
	0–34	5.2 ± 5.1 (22)	37.3 ± 10.8 (22)
	35–49	8.8 ± 0.8 (1307)	11.7 ± 0.9 (1345)
	50–64	6.1 ± 0.3 (9607)	10.4 ± 0.4 (8622)***
	65–79	4.4 ± 0.2 (11881)	6.3 ± 0.3 (12019)***
	80+	2.7 ± 0.5 (2760)	2.8 ± 0.4 (3023)

Data are means ± standard error of the mean, with number of patients in parentheses. Abbreviations: mo, month; RSR, relative survival rate; SEM, standard error of the mean. *p < 0.01, ** *p* < 0.001, and *** *p* < 0.0001 for comparisons with the Male group.

In addition, survival superiority in females was more evident in some age groups, as illustrated in Table 2 and Fig. 3. Significantly higher 12-month RSRs in females were seen in patients aged 50–64 years in each decade (44.9% vs. 35.1% in 1983–1992, 45.7% vs. 35.6% in 1993–2002, 45.1% vs. 35.4% in 2003–2012; *p* < 0.0001 for each). In addition, the age-dependent survival difference between sexes was also observed in the 35–49 and 65–79 age groups. A similar trend in RSR differences between the sexes was noted in the 24- and 36-month RSRs (Suppl. Table S2 and Suppl. Figure S1). The significant age-dependent RSR differences between the sexes were confirmed by Kaplan-Meier curves in most age groups, except for the 0–34 year group (*p* = 0.0585 for group 0–34 years, *p* = 0.0025 for group 80+ years and *p* < 0.0001 for the rest, Fig. 3b).

Furthermore, age, SES, and sex were independent predictors for overall survival over the three decades according to Cox regression analyses (*p* < 0.001) (Suppl. Table S3). The hazard ratios of all of the variables remained stable in each decade and for the three decades combined.

### Survival by race and SES

**Figure 4:**
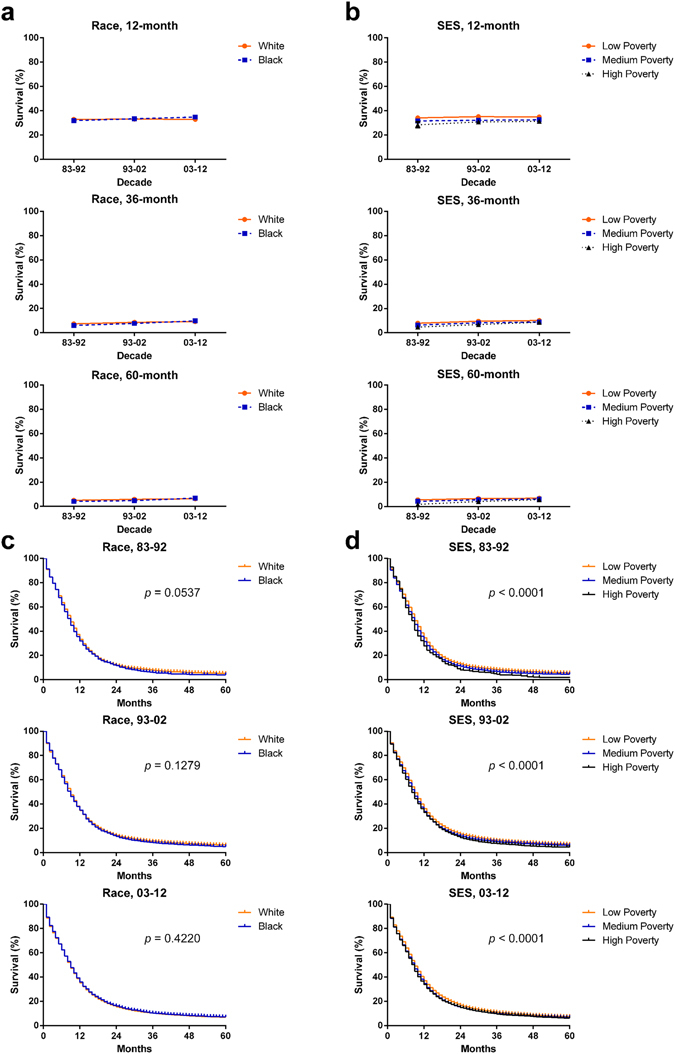
12-month, 36-month and 60-month relative survival rates by race (**a**) and SES/county-level poverty rates (**b**) and Kaplan-Meier survival analyses according to race (**c**) and SES/county-level poverty rates (**d**) for patients with SCLC at 18 SEER sites from 1983 to 2012.

The 12-month RSRs remained relatively stable, whereas a slight improvement could be seen in the 36- and 60-month RSRs in all races and SES groups. Whites shared similar RSRs with Blacks in each decade (Fig. 4a, Table 3 and Suppl. Table S4). Kaplan-Meier survival analyses showed that the survival disparity between Whites and Blacks continued to diminish each decade with increasing *p* values from 0.0537 to 0.1279 to 0.4220 (Fig. 4c). After classifying all patients, including Hispanic or those with a non-Hispanic status, by racial records in the SEER database, Hispanic patients, as a major minority, shared similar survival with non-Hispanic patients in each decade and the three decades combined (Suppl. Figure S4). In terms of SES, the highest SES was found in the low-poverty group, with the lowest in the high-poverty group (Fig. 4b, Table 4, and Suppl. Table S5). In addition, statistically significant differences in survival among the three SES groups were found each decade (*p* < 0.0001 for each); more importantly, the survival disparities narrowed each decade (Fig. 4d). Interestingly, the percentage of low-poverty patients in the Whites group was dramatically higher than in the Blacks group (43.8% vs. 17.5%), but the percentage of medium-poverty individuals was higher in the Black group over the three decades (71.5% vs. 48.0%; Suppl. Figure S5; Suppl. Table S6). The variables race and SES were related, with Spearman rank correlation coefficients of 0.121 and *p* < 0.001. Indeed, the survival difference between Whites and Blacks reflected their different distribution in SES.

**Table 3:** 12-month and 60-month relative survival rates of SCLC patients according to race, age group, and calendar period from 1983 to 2012 at 18 SEER sites.

Decade	Age Group	Race		
		White	Black	Other
83–92	12-Mo RS			
	Total	32.9 ± 0.4 (18537)	31.9 ± 1.2 (1635)	33.2 ± 1.8 (718)
	0–34	35.0 ± 10.7 (20)	54.7 ± 15.0 (11)	0.0 ± 0.0 (1)
	35–49	44.2 ± 1.6 (941)	39.2 ± 4.0 (149)	45.1 ± 7.9 (41)
	50–64	39.4 ± 0.6 (7009)	34.7 ± 1.8 (719)	40.7 ± 2.9 (292)
	65–79	29.0 ± 0.5 (9283)	29.2 ± 1.8 (688)	28.2 ± 2.5 (335)
	80+	17.3 ± 1.1 (1284)	8.2 ± 3.5 (68)	13.1 ± 5.0 (49)
	60-Mo RS			
	Total	5.0 ± 0.2 (18537)	4.1 ± 0.5 (1635)	4.0 ± 0.8 (718)
	0–34	0.0 ± 0.0 (20)	18.3 ± 11.7 (11)	0.0 ± 0.0 (1)
	35–49	6.2 ± 0.8 (941)	3.5 ± 1.5 (149)	10.1 ± 4.8 (41)
	50–64	6.7 ± 0.3 (7009)	4.9 ± 0.9 (719)	4.8 ± 1.3 (292)
	65–79	4.0 ± 0.2 (9283)	3.3 ± 0.8 (688)	2.9 ± 1.0 (335)
	80+	2.0 ± 0.5 (1284)	0.0 ± 0.0 (68)	0.0 ± 0.0 (49)
93–02	12-Mo RS			
	Total	33.1 ± 0.3 (29313)	33.4 ± 0.9 (2769)	35.4 ± 1.3 (1499)
	0–34	50.1 ± 7.7 (42)	66.7 ± 27.2 (3)	0.0 ± 0.0 (4)
	35–49	44.8 ± 1.2 (1596)	43.0 ± 3.3 (227)	45.3 ± 5.5 (83)
	50–64	40.4 ± 0.5 (9774)	36.5 ± 1.5 (1085)	46.4 ± 2.4 (431)
	65–79	30.0 ± 0.4 (14965)	31.8 ± 1.4 (1264)	32.2 ± 1.7 (806)
	80+	18.1 ± 0.7 (2936)	12.8 ± 2.6 (190)	18.4 ± 3.1 (175)
	60-Mo RS			
	Total	5.9 ± 0.1 (29313)	4.8 ± 0.4 (2769)*	7.9 ± 0.7 (1499)***
	0–34	14.3 ± 5.4 (42)	0.0 ± 0.0 (3)	0.0 ± 0.0 (4)
	35–49	9.1 ± 0.7 (1596)	7.1 ± 1.8 (227)	11.1 ± 3.5 (83)
	50–64	7.8 ± 0.3 (9774)	5.7 ± 0.7 (1085)	11.3 ± 1.6 (431)
	65–79	4.8 ± 0.2 (14965)	4.1 ± 0.6 (1264)	6.7 ± 0.9 (806)
	80+	2.9 ± 0.4 (2936)	0.9 ± 0.9 (190)	3.3 ± 1.6 (175)
03–12	12-Mo RS			
	Total	32.9 ± 0.2 (44105)	34.8 ± 0.7 (4413)*	35.8 ± 1.1 (2010)*
	0–34	62.1 ± 8.3 (35)	100.0 ± 0.0 (7)	100.0 ± 0.0 (1)
	35–49	45.2 ± 1.1 (2248)	44.1 ± 2.7 (334)	57.2 ± 6.2 (63)
	50–64	39.7 ± 0.4 (15698)	40.6 ± 1.2 (1864)	44.7 ± 2.0 (638)
	65–79	30.0 ± 0.3 (21049)	30.7 ± 1.1 (1828)	34.5 ± 1.5 (991)*
	80+	17.7 ± 0.6 (5075)	15.4 ± 2.0 (380)	16.9 ± 2.2 (317)
	60-Mo RS			
	Total	6.3 ± 0.1 (44105)	6.9 ± 0.5 (4413)	7.1 ± 0.7 (2010)
	0–34	12.5 ± 6.9 (35)	27.0 ± 21.6 (7)	0.0 ± 0.0 (1)
	35–49	10.0 ± 0.7 (2248)	10.4 ± 1.8 (334)	17.7 ± 5.0 (63)
	50–64	8.0 ± 0.2 (15698)	8.4 ± 0.7 (1864)	10.4 ± 1.3 (638)
	65–79	5.4 ± 0.2 (21049)	5.3 ± 0.6 (1828)	5.3 ± 0.9 (991)
	80+	2.7 ± 0.3 (5075)	3.7 ± 1.2 (380)	2.9 ± 1.2 (317)

Data are means ± standard error of the mean, with number of patients in parentheses. Abbreviations: Mo, month; RS, relative survival; SEM, standard error of the mean. *p < 0.01, **p < 0.001, and ***p < 0.0001 for comparisons with the White group.

**Table 4:** 12-month and 60-month relative survival rates of SCLC patients according to SES, age group, and calendar period from 1983 to 2012 at 18 SEER sites.

Decade	Age Group	SES		
		Low Poverty	Medium Poverty	High Poverty
83–92	12-Mo RS			
	Total	34.1 ± 0.5 (11460)	31.6 ± 0.5 (9074)**	28.4 ± 2.4 (366)
	0–34	42.9 ± 10.8 (21)	36.4 ± 14.5 (11)	0.0 ± 0.0 (0)
	35–49	46.7 ± 2.0 (605)	40.3 ± 2.2 (513)	37.6 ± 12.1 (16)
	50–64	40.9 ± 0.7 (4393)	37.0 ± 0.8 (3481)**	32.6 ± 3.9 (149)
	65–79	29.5 ± 0.6 (5682)	28.4 ± 0.7 (4454)	25.6 ± 3.4 (174)
	80+	17.6 ± 1.5 (759)	15.6 ± 1.5 (615)	16.1 ± 7.4 (27)
	60-Mo RS			
	Total	5.5 ± 0.2 (11460)	4.3 ± 0.2 (9074)***	1.8 ± 0.7 (366)*
	0–34	0.0 ± 0.0 (21)	18.3 ± 11.7 (11)	0.0 ± 0.0 (0)
	35–49	6.7 ± 1 (605)	5.2 ± 1.0 (513)	0.0 ± 0.0 (16)
	50–64	7.0 ± 0.4 (4393)	6.0 ± 0.4 (3481)	1.4 ± 1.0 (149)
	65–79	4.5 ± 0.3 (5682)	3.3 ± 0.3 (4454)*	2.6 ± 1.3 (174)
	80+	2.7 ± 0.8 (759)	0.8 ± 0.5 (615)	0.0 ± 0.0 (27)
93–02	12-Mo RS			
	Total	35.1 ± 0.4 (13882)	32.1 ± 0.4 (17506)***	30.8 ± 1.0 (2228)***
	0–34	45.9 ± 10.2 (24)	50.1 ± 10.7 (22)	33.4 ± 27.2 (3)
	35–49	46.3 ± 1.8 (783)	44.1 ± 1.6 (957)	39.1 ± 3.8 (167)
	50–64	42.7 ± 0.7 (4624)	38.7 ± 0.6 (5854)***	37.0 ± 1.7 (821)*
	65–79	32.0 ± 0.6 (7049)	29.1 ± 0.5 (8956)**	27.1 ± 1.4 (1053)*
	80+	18.2 ± 1.1 (1402)	17.7 ± 1.0 (1717)	16.6 ± 2.9 (184)
	60-Mo RS			
	Total	6.6 ± 0.2 (13882)	5.6 ± 0.2 (17506)**	4.2 ± 0.4 (2228)***
	0–34	12.5 ± 6.8 (24)	13.7 ± 7.3 (22)	0.0 ± 0.0 (3)
	35–49	10.7 ± 1.1 (783)	8.3 ± 0.9 (957)	4.9 ± 1.7 (167)
	50–64	8.7 ± 0.4 (4624)	7.3 ± 0.4 (5854)	5.5 ± 0.8 (821)*
	65–79	5.3 ± 0.3 (7049)	4.7 ± 0.2 (8956)	3.4 ± 0.6 (1053)
	80+	3.2 ± 0.6 (1402)	2.6 ± 0.5 (1717)	1.7 ± 1.2 (184)
03–12	12-Mo RS			
	Total	34.9 ± 0.4 (17819)	32.5 ± 0.3 (26739)***	31.3 ± 0.6 (6045)***
	0–34	83.4 ± 10.8 (12)	67.1 ± 9.6 (25)	57.2 ± 18.7 (7)
	35–49	50.4 ± 1.7 (905)	42.7 ± 1.4 (1346)**	43.2 ± 2.5 (401)
	50–64	42.4 ± 0.6 (6226)	39.2 ± 0.5 (9622)***	36.9 ± 1.0 (2379)***
	65–79	31.8 ± 0.5 (8442)	29.9 ± 0.4 (12690)*	27.5 ± 0.9 (2765)***
	80+	18.7 ± 0.9 (2234)	17.0 ± 0.7 (3056)	14.8 ± 1.7 (493)
	60-Mo RS			
	Total	6.9 ± 0.2 (17819)	6.2 ± 0.2 (26739)	5.7 ± 0.3 (6045)*
	0–34	16.7 ± 10.8 (12)	7.2 ± 6.6 (25)	57.2 ± 18.7 (7)
	35–49	12.3 ± 1.2 (905)	9.4 ± 0.9 (1346)	8.8 ± 1.6 (401)
	50–64	9.1 ± 0.4 (6226)	7.9 ± 0.3 (9622)	6.6 ± 0.6 (2379)**
	65–79	5.5 ± 0.3 (8442)	5.3 ± 0.2 (12690)	4.9 ± 0.5 (2765)
	80+	3.6 ± 0.6 (2234)	2.2 ± 0.4 (3056)	2.2 ± 1 (493)

Data are means ± standard error of the mean, with number of patients in parentheses. Abbreviations: Mo, month; RS, relative survival; SEM, standard error of the mean. *p < 0.01, **p < 0.001, and ***p < 0.0001 for comparisons with the Low Poverty group.

## Discussion

Here, we demonstrated that the incidence of SCLC declined each decade in terms of the total patient population and each patient group stratified by age, sex, race or SES. Long-term survival improved slightly, especially in the first two decades, despite the relatively stable median survival time. Moreover, the survival gaps among races or SES groups diminished each decade.

### The incidence and etiology of SCLC

The incidence of SCLC declined in the total population and most age groups, except for the 80+ age group, in which the incidence remained relatively stable. Risk factors for lung cancer mainly included smoking, air pollution, and exposure to carcinogenic chemicals, such as asbestos, arsenic, radon, and polycyclic aromatic hydrocarbons^2, 10, 27^. The lung cancer rates and trends reflected the trends of tobacco consumption^28–30^. In the United States, smoking rates and lung cancer occurrence declined markedly after increased public awareness of the harm of smoking following the United States Surgeon General’s Reports released in 1964 and deployment of comprehensive tobacco control programs^31–33^. Recent studies found that TP53, RB1, NOTCH, MYC and PI3K are aberrantly mutated in SCLC samples; however, well-established etiological factors for SCLC, like the EGFR-mutation in NSCLC, have not been identified^34–37^.

### Current trends and challenges in SCLC survival

Although the long-term survival of patients with SCLC improved each decade, long-term survival remained at an extremely low level, indicating a pressing need for improvements in the of management of SCLC. In addition, despite the significant changes observed in survival time over the three decades, the absolute increments in each decade were marginal in both the total population and each patient group stratified. Several factors were responsible for the slight survival improvement over the three decades. First, unlike cases in NSCLC, attempts of using novel agents, including pravastatin and targeted therapy, for SCLC failed, and radiotherapy plus platinum-based chemotherapy with etoposide has been the standard care since the early 1980s, but an efficacy plateau has been reached^5–8, 38–40^. Earlier administration of radiotherapy can marginally improve overall survival^41^. Second, based on our data, most cases were diagnosed as distant cases, with 62%, 64% and 71% in this category in each decade, respectively, indicating the exigence of improving clinical awareness of early detection and detection techniques (Suppl. Table S7). Early screening by low-dose computed topography may help identify early stage lung cancer and thereby benefit patients; however, this screening has not been widely adapted^42–45^. Third, the development of targeted therapy and clinical staging was halted by a scarcity of well-elucidated driver mutations and pathological characteristics. In addition, EGFR-TKI,BCR-ABL TKIs, mTOR inhibitors and anti-angiogenesis treatments have not received approval for SCLC due to a lack of clinical efficiency^9, 10^. The slight improvement in long-term survival may be due to developments in second line chemotherapy and hospice care. Recently, preliminary data from clinical trials on CTLA4 and PD-1 inhibitors have demonstrated promising efficiency at treating SCLC, with durable and manageable AEs^5^. In addition, 22.5% of lung cancer cases fail to receive timely treatment, and great importance should therefore be attached to the timeliness of receiving treatment^46^.

### Sexual disparities in SCLC

In sharp contrast to the markedly declined incidence trend in males, the incidence in females remained stable across the three decades. Males had more space to decline due to increased tobacco consumption, and females showed a weaker response to tobacco cessation programs^47–49^. Despite the stable 12-month RSRs, we observed a slightly improving long-term survival in both sexes, with survival superiority in females, indicating an intrinsic genomic differences between the sexes.

### Racial disparities in SCLC

The incidence of SCLC in all races decreased each decade, and the incidence gap between the Black and Other groups continued to narrow over the three decades. As demonstrated by the Kaplan-Meier curves, no significant survival difference existed between Whites and Blacks, and the survival gap among them continued to narrowed in each decade. Furthermore, the outcomes for both African-Americans and non-African-Americans were similar when equivalent therapies were offered^50^. Interestingly, a higher proportion of White patients were classified as low-poverty compared to Black patients. Similarly, the narrowing survival disparities in both races and SES groups may have been due to differing SES distributions between Blacks and Whites since financially disadvantaged patients were believed to be less likely to receive medical care, ultimately leading to shorter survival. In addition, Hispanics, a major minority, showed similar survival with non-Hispanics in each decade and the three decades combined.

### SCLC Disparities among the SES groups

There were differences in the incidence of SCLC among the various SES groups, with the highest incidence in the low- and medium-poverty groups. Lower incidence in the high-poverty group could be partly due to a lower consumption of cigarettes and living in less industrialized regions, keeping them away from air pollution. The survival differed among different SES groups, however, and the survival gaps among the various SES groups continued to narrow each decade (*p* < 0.0001 for each). This narrowing trend may be attributed to the following reasons: first, the development of health care systems narrows the survival disparities among different SES groups by covering more patients, and second, the standard first-line chemotherapy regimen and the radiotherapy, which are applicable to most SCLC cases, were financially available to most patients. However, the cost of SCLC treatment is considerable as was the financial heterogeneity inside patient groups, which may have led to the aforementioned survival disparity^12^. Therefore, healthcare regulations to compensate financially disadvantaged patients may help fill the gaps among SES groups.

In this study, we did not divide patients by stage because this was outside the scope of this study and the staging systems (both TNM and VALSG) have changed over time. In addition, the patient data from selected SEER registry sites only reflect the tendencies of selected areas, and therefore, the results and conclusions should be interpreted with caution when being applied to other areas. Furthermore, this study may be affected by bias and errors if there is any under-registration or misclassification in the SEER database^51^.

In general, this study demonstrated the declining incidence and relatively stable survival rates of SCLC. However, the medical burden brought on by SCLC remains significant, indicating the urgency not only for early detection and prevention but also for developing novel therapies. In addition, the survival disparities demonstrated in this study imply that there is heterogeneity in SCLC. This study may help in the design of better clinical trials by balancing these disparities and ultimately improving the clinical treatment of SCLC. Furthermore, the trends shown here highlight the need for developing novel agents by clarifying genetic patterns and may assist politicians in passing legislation to rectify disparities among SES groups.

## Materials and Methods

### Data extraction from SEER database

All cases of patients diagnosed with SCLC between 1983 and 2012 were accessed from the SEER database, a database maintained by the National Cancer Institute, covering approximately 28% of the U.S. population^52^. In addition, incidence data and survival data were obtained from nine original registry sites and 18 registry sites, respectively. SEER*Stat version 8.3.2 software was used for all data collection.

### Categorized SCLC cases across three decades

Patients were included on the basis of the SEER site recode using the International Classification of Diseases for Oncology, Third Edition (2008), Lung and Bronchus (C34.0–C34.9). We accessed only the cases diagnosed between 1983 and 2012. SCLC cases were histologically defined by the following International Classification of Diseases for Oncology, Third Edition, histology codes for malignant cases: 8041/2 (Small cell carcinoma *in situ*), 8041/3 (Small cell carcinoma, NOS), 8042/3 (Oat cell carcinoma), 8043/3 (Small cell carcinoma, fusiform cell), 8044/3 (Small cell carcinoma, intermediate cell), and 8045/3 (Combined small cell carcinoma). We excluded cases of SCLC that had been identified by an autopsy or a death certificate.

We analyzed incidence and survival data in each decade between 1983 and 2012. Furthermore, patient cases were classified by race, sex, age, and SES. We defined SES levels as published previously; briefly, SES levels, on the basis of the county poverty rate, were divided into three categories^25^. In addition, all patients included were classified as either Hispanic or non-Hispanic based on the racial record in the SEER database, and their survival was compared, as Hispanics are a major minority in U.S.

### Incidence and survival trends

Incidence data were calculated per 100,000 persons and RSR was restricted to the deaths owing directly to SCLC^25^. The differences between Kaplan-Meier curves were evaluated by a two-tailed log-rank test.

### Cox regression and Spearman rank correlation analyses

The Others group was excluded from Cox regression analysis due to disparities within the group. In addition, the original high-poverty group and medium-poverty group were merged as a novel “med-high-poverty group”. Ages were divided into five groups, as previously discussed. We performed Spearman rank correlation analyses because race and SES were not normally distributed. Stata MP 14 software (Stata Corp) was used for Cox regression analyses and Spearman rank correlation analyses. A two-tailed *p* value of <0.01 was considered statistically significant.

## Electronic supplementary material

Supplementary file
