# Supplementary material for: Survival changes in patients with small cell lung cancer and disparities between different sexes, socioeconomic statuses and ages
Source: Sci Rep. 2017 May 2;7:1339. doi: 10.1038/s41598-017-01571-0 (PMC5431017; doi:10.1038/s41598-017-01571-0)
Supplement: Supplementary file 1 — Supplementary file [file 41598_2017_1571_MOESM1_ESM.doc]

**Survival changes in patients with small cell lung cancer and disparities between different sexes, socioeconomic statuses and ages**

Shuncong Wang^1#^ M.D., Jianjun Tang^2#^ M.D. & Ph.D., Tiantian Sun^3^ M.D. & Ph.D., Xiaobin Zheng^4^ M.D. & Ph.D., Jie Li^5^ M.D. & Ph.D., Hongliu Sun^6^ M.D. & Ph.D., Xiuling Zhou^1^ M.D., Cuiling Zhou^1^ M.D., Hongyu Zhang^1^ M.D. & Ph.D., Zhibin Cheng^1^ M.D., Haiqing Ma^1*^ M.D. & Ph.D., Huanhuan Sun^1*^ M.D. & Ph.D.

^1^Department of Oncology, The Fifth Affiliated Hospital of Sun Yat-sen University, Zhuhai, Guangdong 519000, China;

^2^Department of Gastroenterology, Cancer Hospital of Jiangxi Province, Nanchang, Jiangxi 330029, China;

^3^Department of Hematology, The First Affiliated Hospital of Sun Yat-sen University, Guangzhou, Guangdong 510080, China;

^4^Department of Respiration, The Fifth Affiliated Hospital of Sun Yat-sen University, Zhuhai, Guangdong 519000, China;

^5^Department of Breast and Thyroid Surgery, The First Affiliated Hospital of Sun Yat-sen University, Guangzhou, Guangdong 510080, China;

^6^Department of Pathology, University of Michigan, Ann Arbor, MI 48201， USA

^#^These authors contributed equally to this work.

^*^correspondence to:

Haiqing Ma M.D. & Ph.D., Department of Oncology, The Fifth Affiliated Hospital of Sun Yat-sen University, NO. 52 Meihua East Rd., Zhuhai, Guangdong 519000, China; (mahaiqing@mail.sysu.edu.cn) Work Telephone: +86-0756-2528656; FAX: +86-0756-2528210

Huanhuan Sun M.D. & Ph.D., Department of Oncology, The Fifth Affiliated Hospital of Sun Yat-sen University, NO. 52 Meihua East Rd., Zhuhai, Guangdong 519000, China; (sunhuanh3@mail.sysu.edu.cn) Work Telephone: +86-0756-2528604; FAX: +86-0756-2528210


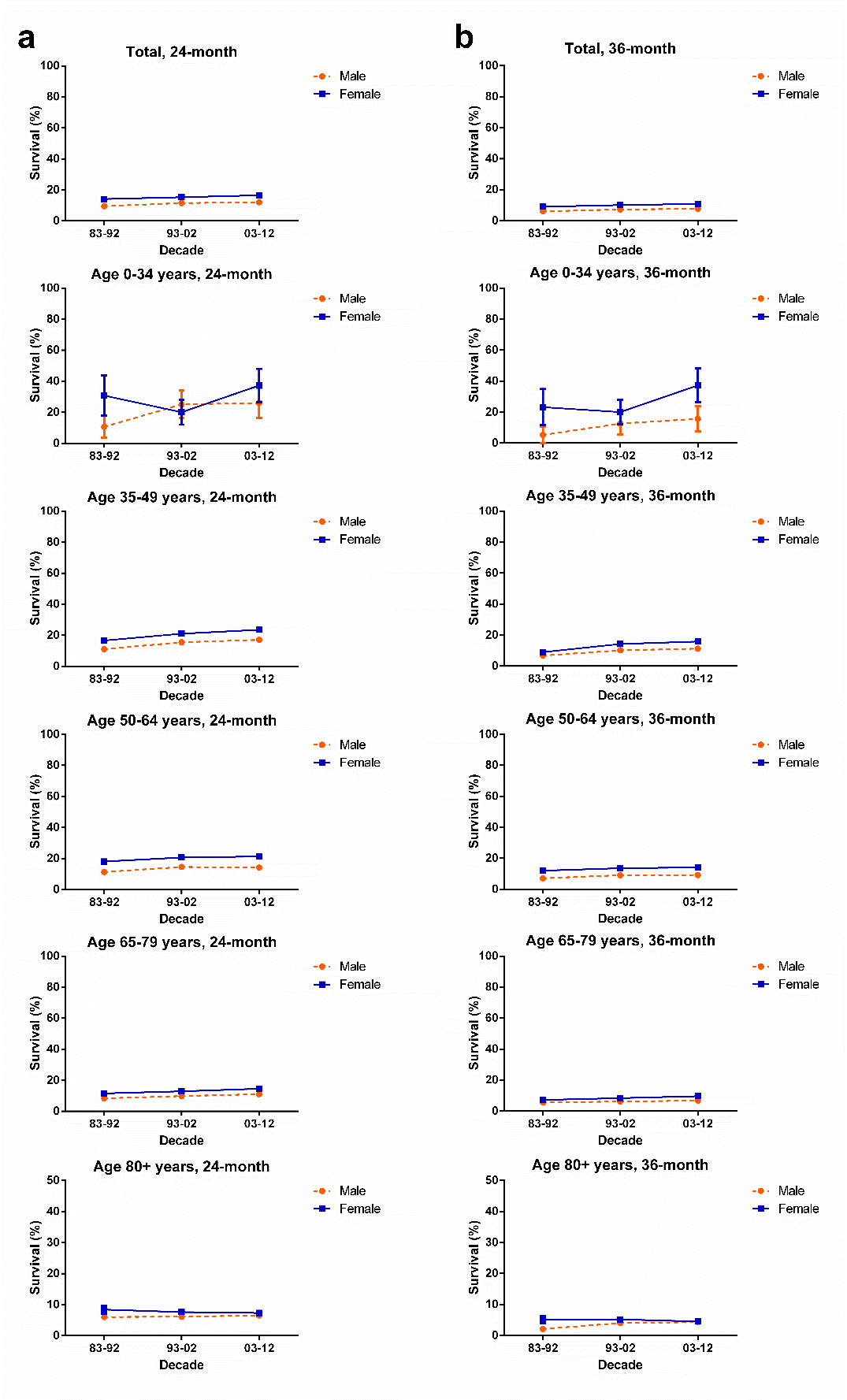
**Supplementary Figure S1**

**Supplementary Figure S1.** 24-month (a) and 36-month (b) relative survival rates for patients with SCLC at 18 SEER sites from 1983 to 2012 according to sex and age group (total and ages 0-34, 35-59, 50–64, 65-59 and 80+ years).


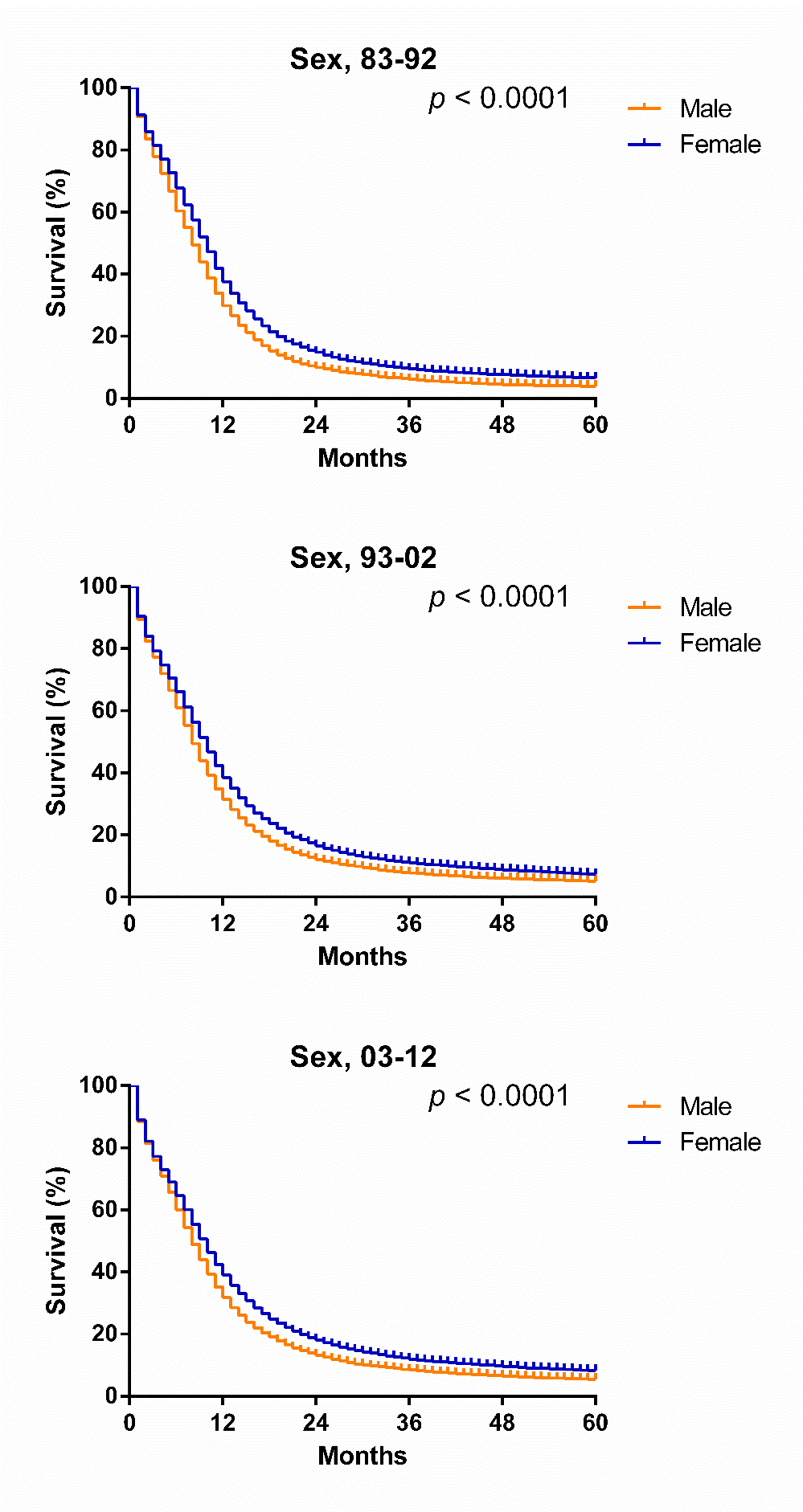
**Supplementary Figure S2**

**Supplementary Figure S2.** Kaplan-Meier survival analyses for patients with SCLC from 18 SEER sites from 1983 to 2012 according to sex and calendar period.


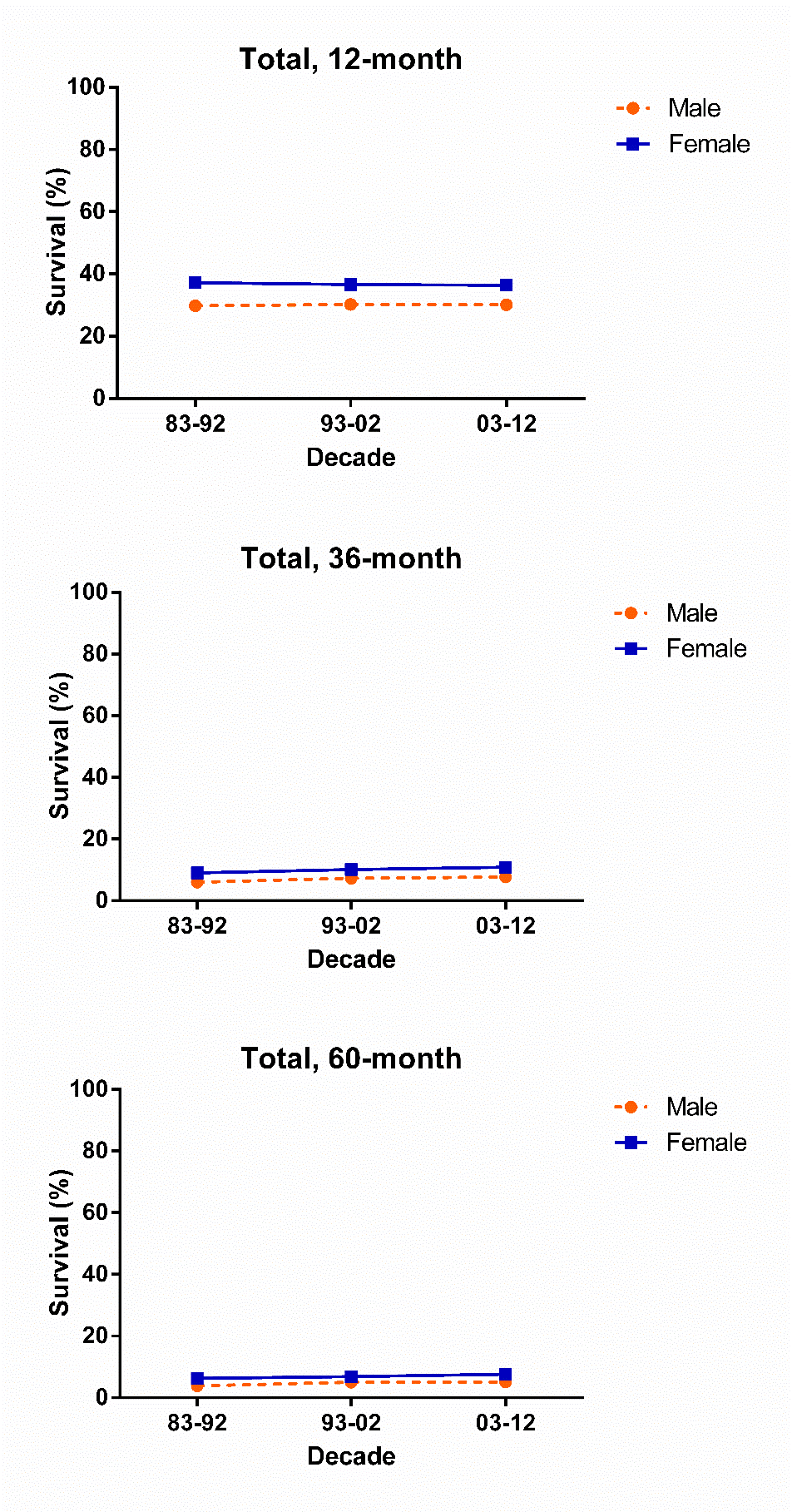
**Supplementary Figure S3**

**Supplementary Figure S3.** 12-month, 36-month, and 60-month relative survival rates for patients with SCLC from 1983 to 2012 at 18 SEER sites by sex and calendar period.


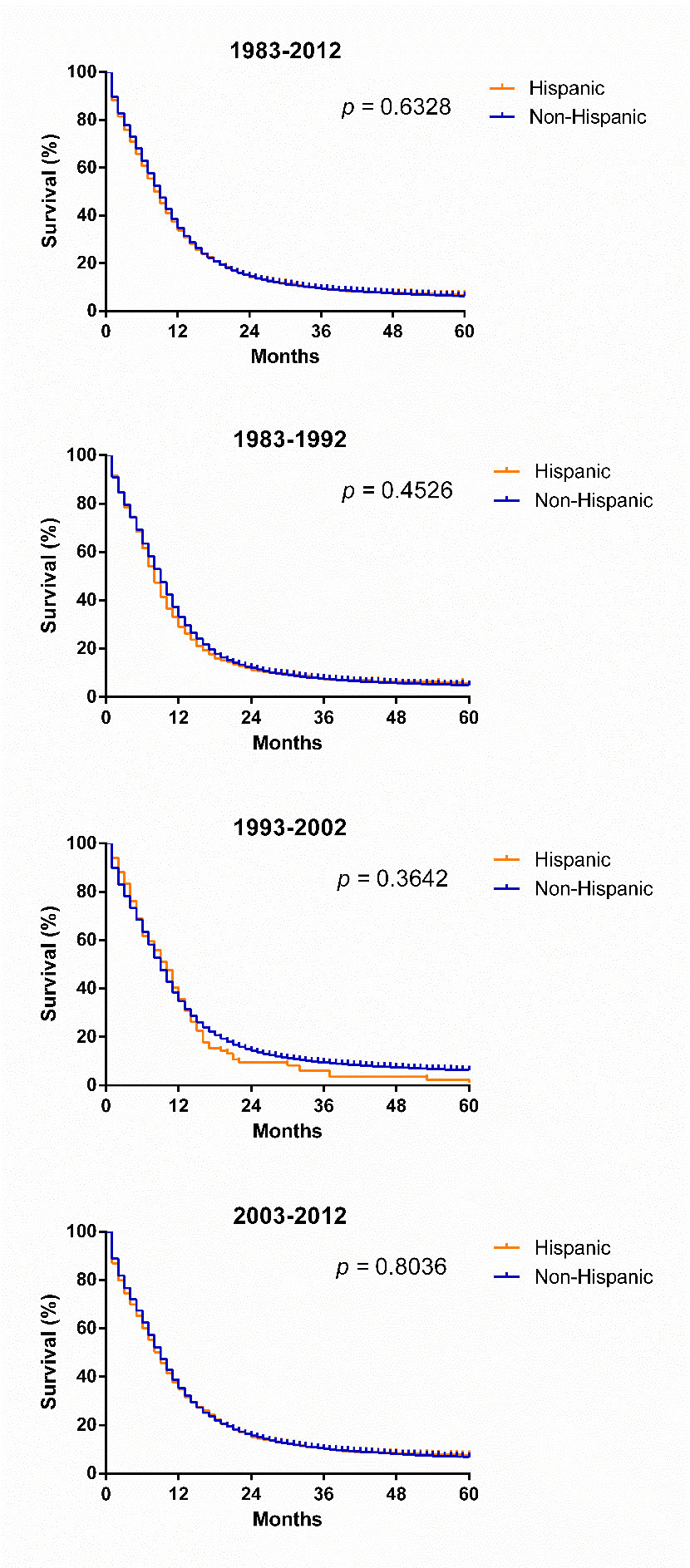
**Supplementary Figure S4**

**Supplementary Figure S4.** Kaplan-Meier survival analyses for patients with SCLC from 18 SEER sites from 1983 to 2012 by race (Hispanic or non-Hispanic) and calendar period.

**Supplementary Figure S5**

**
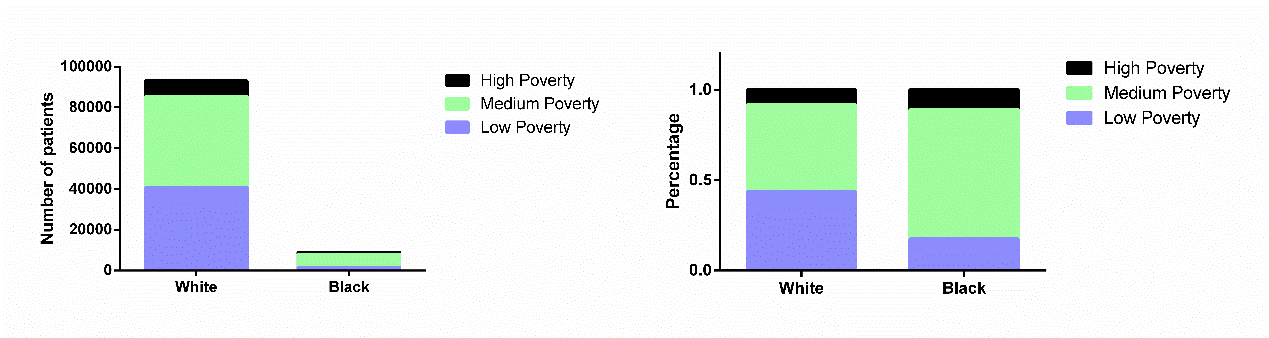
**

**Supplementary Figure S5.** Distribution of socioeconomic status by race for patients with SCLC at 18 SEER sites between 1983 and 2012. Percentage (a) and number (b) of patients with SCLC in low-poverty, medium-poverty, and high-poverty groups.

**Supplementary Table S1**. Incidence of SCLC according to age group and decade within sex, SES, and race groups from 1983 to 2012 at the nine original SEER sites. Data are incidence per 100,000 people by year of diagnosis, with the number of patients in parentheses.

|  | | **Age**  **Group** | **Decade** | | |
| --- | --- | --- | --- | --- | --- |
| **Variable** | |  | **1983-1992** | **1993-2002** | **2003-2012** |
| Total |  |  |  |  |  |
|  |  | Total | 9.6 (20380) | 7.8 (18920) | 5.8 (16920) |
|  |  | 0-34 | 0.0 (31) | 0.0 (28) | 0.0 (10) |
|  |  | 35-49 | 2.6 (1118) | 1.8 (1075) | 1.3 (832) |
|  |  | 50-64 | 24.4 (7863) | 17.4 (6288) | 11.5 (5985) |
|  |  | 65-79 | 48.7 (10022) | 43.0 (9693) | 33.1 (7927) |
|  |  | 80+ | 22.9 (1346) | 23.0 (1836) | 22.0 (2166) |
| Sex | Male |  |  |  |  |
|  |  | Total | 12.9 (12029) | 9.2 (9874) | 6.4 (8397) |
|  |  | 0-34 | 0.0 (18) | 0.0 (12) | 0.0 (5) |
|  |  | 35-49 | 3.3 (684) | 1.9 (566) | 1.2 (399) |
|  |  | 50-64 | 30.2 (4652) | 19.4 (3387) | 12.2 (3098) |
|  |  | 65-79 | 66.6 (5860) | 50.7 (4996) | 35.8 (3882) |
|  |  | 80+ | 42.4 (815) | 33.2 (913) | 27.8 (1013) |
|  | Female |  |  |  |  |
|  |  | Total | 7.1 (8351) | 6.8 (9046) | 5.4 (8523) |
|  |  | 0-34 | 0.0 (13) | 0.0 (16) | 0.0 (5) |
|  |  | 35-49 | 2.0 (434) | 1.7 (509) | 1.3 (433) |
|  |  | 50-64 | 19.2 (3211) | 15.5 (2901) | 10.7 (2887) |
|  |  | 65-79 | 35.4 (4162) | 37.1 (4697) | 30.8 (4045) |
|  |  | 80+ | 13.5 (531) | 17.9 (923) | 18.8 (1153) |
| Race | white |  |  |  |  |
|  |  | Total | 9.9 (18086) | 8.2 (16536) | 6.2 (14477) |
|  |  | 0-34 | 0.0 (19) | 0.0 (23) | 0.0 (8) |
|  |  | 35-49 | 2.7 (930) | 1.9 (915) | 1.4 (702) |
|  |  | 50-64 | 25.4 (6873) | 18.5 (5434) | 12.3 (5036) |
|  |  | 65-79 | 50.8 (9030) | 45.4 (8528) | 35.5 (6852) |
|  |  | 80+ | 23.5 (1234) | 23.6 (1636) | 22.8 (1879) |
|  | Black |  |  |  |  |
|  |  | Total | 9.7 (1599) | 7.5 (1482) | 5.4 (1433) |
|  |  | 0-34 | 0.1 (11) | 0.0 (3) | 0.0 (2) |
|  |  | 35-49 | 3.6 (147) | 1.7 (114) | 1.2 (98) |
|  |  | 50-64 | 26.9 (708) | 17.8 (585) | 11.7 (626) |
|  |  | 65-79 | 44.1 (668) | 40.2 (682) | 28.8 (575) |
|  |  | 80+ | 19.2 (65) | 20.2 (98) | 20.9 (132) |
|  | Other |  |  |  |  |
|  |  | Total | 4.8 (683) | 4.1 (886) | 3.1 (987) |
|  |  | 0-34 | 0.0 (1) | 0.0 (2) | 0.0 (0) |
|  |  | 35-49 | 1.1 (38) | 0.8 (46) | 0.4 (30) |
|  |  | 50-64 | 11.3 (278) | 7.5 (263) | 5.3 (311) |
|  |  | 65-79 | 24.3 (319) | 23.3 (473) | 18.4 (492) |
|  |  | 80+ | 16.0 (47) | 18.8 (102) | 16.2 (154) |
| SES | Low Poverty |  |  |  |  |
|  |  | Total | 9.7 (11443) | 7.8 (10834) | 5.8 (10135) |
|  |  | 0-34 | 0.0 (21) | 0.0 (19) | 0.0 (6) |
|  |  | 35-49 | 2.5 (607) | 1.8 (623) | 1.3 (503) |
|  |  | 50-64 | 24.5 (4384) | 17.2 (3561) | 11.3 (3534) |
|  |  | 65-79 | 50.2 (5675) | 43.6 (5544) | 33.3 (4716) |
|  |  | 80+ | 23.0 (756) | 23.4 (1087) | 22.9 (1376) |
|  | Medium Poverty |  |  |  |  |
|  |  | Total | 9.5 (8572) | 7.9 (7676) | 5.9 (6397) |
|  |  | 0-34 | 0.0 (10) | 0.0 (9) | 0.0 (4) |
|  |  | 35-49 | 2.8 (495) | 1.8 (430) | 1.3 (318) |
|  |  | 50-64 | 24.6 (3330) | 18.0 (2603) | 11.9 (2324) |
|  |  | 65-79 | 47.4 (4174) | 42.6 (3928) | 33.2 (3001) |
|  |  | 80+ | 22.7 (563) | 22.5 (706) | 21.0 (750) |
|  | High Poverty |  |  |  |  |
|  |  | Total | 7.3 (363) | 6.4 (408) | 4.7 (384) |
|  |  | 0-34 | 0.0 (0) | 0.0 (0) | 0.0 (0) |
|  |  | 35-49 | 1.6 (16) | 1.5 (22) | 0.7 (11) |
|  |  | 50-64 | 18.6 (148) | 12.6 (123) | 9.1 (125) |
|  |  | 65-79 | 36.3 (172) | 36.5 (220) | 28.1 (208) |
|  |  | 80+ | 21.9 (27) | 23.8 (43) | 16.6 (40) |

Abbreviation: SES, socioeconomic status.

**Supplementary Table S2**. 24-month and 36-month relative survival rates of SCLC patients according to sex, age group, and calendar period from 1983 to 2012 at 18 SEER sites. Data are means ± standard error of the mean, with number of patients in parentheses.

|  |  | **Sex** | | | |
| --- | --- | --- | --- | --- | --- |
| **Decade** | **Age Group** | **Male** | **Female** | | |
| 83-92 | 24-Mo RS |  | |  |  |
|  | Total | 9.5 ± 0.3 (12341) | | 14.1 ± 0.4 (8561)*** |  |
|  | 0-34 | 10.6 ± 7.1 (19) | | 30.8 ± 12.8 (13) |  |
|  | 35-49 | 11.0 ± 1.2 (698) | | 16.6 ± 1.8 (436)* |  |
|  | 50-64 | 11.3 ± 0.5 (4759) | | 18.0 ± 0.7 (3265)*** |  |
|  | 65-79 | 8.4 ± 0.4 (6026) | | 11.5 ± 0.5 (4285)*** |  |
|  | 80+ | 6.0 ± 0.9 (839) | | 8.4 ± 1.3 (562) |  |
|  | 36-Mo RS |  | |  |  |
|  | Total | 6.0 ± 0.2 | | 9.0 ± 0.3*** |  |
|  | 0-34 | 5.3 ± 5.2 | | 23.2 ± 11.7 |  |
|  | 35-49 | 6.8 ± 1.0 | | 9.0 ± 1.4 |  |
|  | 50-64 | 7.1 ± 0.4 | | 12 ± 0.6*** |  |
|  | 65-79 | 5.5 ± 0.3 | | 7.2 ± 0.4** |  |
|  | 80+ | 2.1 ± 0.6 | | 5.1 ± 1.1* |  |
| 93-02 | 24-Mo RS |  | |  |  |
|  | Total | 11.5 ± 0.2 (17657) | | 15.3 ± 0.3 (15961)*** |  |
|  | 0-34 | 25.1 ± 8.9 (24) | | 20.0 ± 8.0 (25) |  |
|  | 35-49 | 15.5 ± 1.1 (1045) | | 21.0 ± 1.4 (862)* |  |
|  | 50-64 | 14.5 ± 0.5 (6139) | | 20.6 ± 0.6 (5161)*** |  |
|  | 65-79 | 9.8 ± 0.3 (8813) | | 12.9 ± 0.4 (8246)*** |  |
|  | 80+ | 6.1 ± 0.7 (1636) | | 7.6 ± 0.7 (1667) |  |
|  | 36-Mo RS |  | |  |  |
|  | Total | 7.3 ± 0.2 | | 10.1 ± 0.2*** |  |
|  | 0-34 | 12.6 ± 6.8 | | 20.0 ± 8.0 |  |
|  | 35-49 | 10.2 ± 0.9 | | 14.3 ± 1.2* |  |
|  | 50-64 | 9.1 ± 0.4 | | 13.6 ± 0.5*** |  |
|  | 65-79 | 6.2 ± 0.3 | | 8.4 ± 0.3*** |  |
|  | 80+ | 4.0 ± 0.6 | | 5.2 ± 0.6 |  |
| 03-12 | 24-Mo RS |  | |  |  |
|  | Total | 12.0 ± 0.2 (25577) | | 16.5 ± 0.2 (25031)*** |  |
|  | 0-34 | 26.0 ± 9.6 (22) | | 37.3 ± 10.8 (22) |  |
|  | 35-49 | 17.0 ± 1.1 (1307) | | 23.5 ± 1.2 (1345)*** |  |
|  | 50-64 | 14.1 ± 0.4 (9607) | | 21.3 ± 0.5 (8622)*** |  |
|  | 65-79 | 11.0 ± 0.3 (11881) | | 14.5 ± 0.3 (12019)*** |  |
|  | 80+ | 6.5 ± 0.5 (2760) | | 7.3 ± 0.5 (3023) |  |
|  | 36-Mo RS |  | |  |  |
|  | Total | 7.7 ± 0.2 | | 10.9 ± 0.2*** |  |
|  | 0-34 | 15.7 ± 8.1 | | 37.3 ± 10.8 |  |
|  | 35-49 | 11.3 ± 0.9 | | 15.9 ± 1.0** |  |
|  | 50-64 | 9.2 ± 0.3 | | 14.1 ± 0.4*** |  |
|  | 65-79 | 6.8 ± 0.3 | | 9.6 ± 0.3*** |  |
|  | 80+ | 4.3 ± 0.5 | | 4.5 ± 0.4 |  |

Abbreviations: Mo, month; RS, relative survival; SEM, standard error of the mean.

**p* < 0.01, ***p* < 0.001, and ****p* < 0.0001

**Supplementary Table S3**. Summary data for Cox regression analyses of survival in patients with SCLC from 1983 to 2012 at 18 SEER sites.

| **Variable** | **Hazard Ratio (95% CI)** | ***p*-value** |
| --- | --- | --- |
| **All 1983-1992**  Univariate |  |  |
| Sex | 0.831 (0.808-0.855) | <0.001 |
| Age | 1.293 (1.267-1.320) | <0.001 |
| Race | 1.031 (0.980-1.084) | =0.238 |
| SES | 1.077 (1.047-1.107) | <0.001 |
| Multivariate |  |  |
| Sex | 0.832 (0.809-0.856) | <0.001 |
| Age | 1.293 (1.267-1.320) | <0.001 |
| SES | 1.077 (1.047-1.107) | <0.001 |
| **All 1993-2002**  Univariate |  |  |
| Sex | 0.872 (0.853-0.891) | <0.001 |
| Age | 1.316 (1.295-1.336) | <0.001 |
| Race | 1.015 (0.976-1.056) | =0.446 |
| SES | 1.080 (1.057-1.105) | <0.001 |
| Multivariate |  |  |
| Sex | 0.864 (0.845-0.883) | <0.001 |
| Age | 1.320 (1.299-1.340) | <0.001 |
| SES | 1.081 (1.057-1.105) | <0.001 |
| **All 2003-2012**  Univariate |  |  |
| Sex | 0.861 (0.845-0.876) | <0.001 |
| Age | 1.314 (1.298-1.331) | <0.001 |
| Race | 0.955 (0.925-0.986) | =0.004 |
| SES | 1.064 (1.044-1.085) | <0.001 |
| Multivariate |  |  |
| Sex | 0.856 (0.840-0.872) | <0.001 |
| Age | 1.318 (1.301-1.334) | <0.001 |
| Race | 0.975 (0.944-1.007) | =0.124 |
| SES | 1.075 (1.054-1.096) | <0.001 |
| **All 1983-2012**  Univariate |  |  |
| Sex | 0.858 (0.847-0.869) | <0.001 |
| Age | 1.310 (1.299-1.322) | <0.001 |
| Race | 0.987 (0.965 -1.009) | =0.236 |
| SES | 1.067 (1.053-1.080) | <0.001 |
| Multivariate |  |  |
| Sex | 0.852 (0.842-0.863) | <0.001 |
| Age | 1.314 (1.302-1.325) | <0.001 |
| SES | 1.070 (1.057-1.084) | <0.001 |

Abbreviations: 95% CI, 95% confidence interval; SES, socioeconomic status.

**Supplementary Table S4**. 24-month and 36-month relative survival rates of SCLC patients according to race, age group, and calendar period from 1983 to 2012 at 18 SEER sites. Data are means ± standard error of the mean, with number of patients in parentheses.

|  |  | **Race** | | |
| --- | --- | --- | --- | --- |
| **Decade** | **Age Group** | **White** | **Black** | **Other** |
| 83-92 | 24-Mo RS |  |  |  |
|  | Total | 11.4 ± 0.2 (18537) | 11.4 ± 0.8 (1635) | 12.0 ± 1.2 (718) |
|  | 0-34 | 10.0 ± 6.7 (20) | 36.5 ± 14.6 (11) | 0.0 ± 0.0 (1) |
|  | 35-49 | 13.7 ± 1.1 (941) | 8.9 ± 2.3 (149) | 17.6 ± 6.0 (41) |
|  | 50-64 | 14.1 ± 0.4 (7009) | 13.1 ± 1.3 (719) | 15.3 ± 2.1 (292) |
|  | 65-79 | 9.7 ± 0.3 (9283) | 10.5 ± 1.2 (688) | 9.4 ± 1.6 (335) |
|  | 80+ | 7.2 ± 0.8 (1284) | 5.4 ± 3.0 (68) | 4.6 ± 3.2 (49) |
|  | 36-Mo RS |  |  |  |
|  | Total | 7.4 ± 0.2 | 6.0 ± 0.6 | 6.1 ± 0.9 |
|  | 0-34 | 10.0 ± 6.7 | 18.3 ± 11.7 | 0.0 ± 0.0 |
|  | 35-49 | 8.1 ± 0.9 | 4.8 ± 1.8 | 10.1 ± 4.8 |
|  | 50-64 | 9.3 ± 0.4 | 7.3 ± 1.0 | 7.7 ± 1.6 |
|  | 65-79 | 6.3 ± 0.3 | 5.2 ± 0.9 | 4.8 ± 1.2 |
|  | 80+ | 3.4 ± 0.6 | 1.9 ± 1.9 | 2.6 ± 2.5 |
| 93-02 | 24-Mo RS |  |  |  |
|  | Total | 13.2 ± 0.2 (29313) | 12.7 ± 0.7 (2769) | 16.6 ± 1.0 (1499)** |
|  | 0-34 | 26.2 ± 6.8 (42) | 0.0 ± 0.0 (3) | 0.0 ± 0.0 (4) |
|  | 35-49 | 17.7 ± 1.0 (1596) | 18.7 ± 2.6 (227) | 22.1 ± 4.6 (83) |
|  | 50-64 | 17.3 ± 0.4 (9774) | 14.7 ± 1.1 (1085) | 23.6 ± 2.1 (431)* |
|  | 65-79 | 11.2 ± 0.3 (14965) | 10.8 ± 0.9 (1264) | 14.0 ± 1.3 (806) |
|  | 80+ | 6.8 ± 0.5 (2936) | 5.8 ± 1.9 (190) | 9.2 ± 2.4 (175) |
|  | 36-Mo RS |  |  |  |
|  | Total | 8.6 ± 0.2 | 7.7 ± 0.5 | 10.6 ± 0.8 |
|  | 0-34 | 19.1 ± 6.1 | 0.0 ± 0.0 | 0.0 ± 0.0 |
|  | 35-49 | 12.2 ± 0.8 | 10.8 ± 2.1 | 13.5 ± 3.8 |
|  | 50-64 | 11.2 ± 0.3 | 8.9 ± 0.9 | 15.8 ± 1.8* |
|  | 65-79 | 7.3 ± 0.2 | 6.7 ± 0.8 | 8.6 ± 1.0 |
|  | 80+ | 4.6 ± 0.4 | 3.5 ± 1.5 | 5.6 ± 1.9 |
| 03-12 | 24-Mo RS |  |  |  |
|  | Total | 14.1 ± 0.2 (44105) | 15.2 ± 0.6 (4413) | 16 ± 0.9 (2010) |
|  | 0-34 | 22.5 ± 7.3 (35) | 71.6 ± 17.1 (7)* | 0.0 ± 0.0 (1) |
|  | 35-49 | 20.1 ± 0.9 (2248) | 20.2 ± 2.3 (334) | 26.5 ± 5.6 (63) |
|  | 50-64 | 17.4 ± 0.3 (15698) | 17.4 ± 0.9 (1864) | 21.6 ± 1.7 (638)* |
|  | 65-79 | 12.6 ± 0.2 (21049) | 13.8 ± 0.9 (1828) | 14.4 ± 1.2 (991) |
|  | 80+ | 7.0 ± 0.4 (5075) | 5.1 ± 1.3 (380) | 7.7 ± 1.7 (317) |
|  | 36-Mo RS |  |  |  |
|  | Total | 9.2 ± 0.1 | 9.8 ± 0.5 | 10.4 ± 0.7 |
|  | 0-34 | 18.7 ± 7.0 | 53.8 ± 20.2 | 0.0 ± 0.0 |
|  | 35-49 | 13.5 ± 0.7 | 12.7 ± 1.9 | 21.2 ± 5.3 |
|  | 50-64 | 11.4 ± 0.3 | 11.7 ± 0.8 | 13.3 ± 1.4 |
|  | 65-79 | 8.1 ± 0.2 | 8.4 ± 0.7 | 9.2 ± 1.0 |
|  | 80+ | 4.3 ± 0.3 | 3.8 ± 1.2 | 6.0 ± 1.5 |

Abbreviations: Mo, month; RS, relative survival; SEM, standard error of the mean.

**p* < 0.01, ***p* < 0.001, and ****p* < 0.0001 for comparisons with the White group.

**Supplementary Table S5**. 24-month and 36-month relative survival rates of SCLC patients according to SES, age group, and calendar period from 1983 to 2012 at 18 SEER sites. Data are means ± standard error of the mean, with number of patients in parentheses.

|  |  | **SES** | | | |  |
| --- | --- | --- | --- | --- | --- | --- |
| **Decade** | **Age Group** | **Low Poverty** | **Medium Poverty** | **High Poverty** | |  |
| 83-92 | 24-Mo RS |  |  | |  | |
|  | Total | 12.2 ± 0.3 (11460) | 10.5 ± 0.3 (9074)*** | | 8.1 ± 1.5 (366) | |
|  | 0-34 | 14.3 ± 7.7 (21) | 27.4 ± 13.5 (11) | | #VALUE! | |
|  | 35-49 | 15.2 ± 1.5 (605) | 11.0 ± 1.4 (513) | | 6.3 ± 6.1 (16) | |
|  | 50-64 | 15.0 ± 0.5 (4393) | 13.1 ± 0.6 (3481) | | 8.3 ± 2.3 (149) | |
|  | 65-79 | 10.3 ± 0.4 (5682) | 9.0 ± 0.4 (4454) | | 8.6 ± 2.2 (174) | |
|  | 80+ | 8.0 ± 1.1 (759) | 5.9 ± 1.1 (615) | | 4.4 ± 4.3 (27) | |
|  | 36-Mo RS |  |  | |  | |
|  | Total | 7.9 ± 0.3 | 6.4 ± 0.3** | | 4.7 ± 1.2 | |
|  | 0-34 | 9.6 ± 6.4 | 18.3 ± 11.7 | | #VALUE! | |
|  | 35-49 | 8.9 ± 1.2 | 6.3 ± 1.1 | | 6.3 ± 6.1 | |
|  | 50-64 | 9.7 ± 0.5 | 8.5 ± 0.5 | | 3.5 ± 1.5 | |
|  | 65-79 | 6.9 ± 0.4 | 5.3 ± 0.4* | | 6.4 ± 2.0 | |
|  | 80+ | 4.2 ± 0.9 | 2.4 ± 0.7 | | 0.0 ± 0.0 | |
| 93-02 | 24-Mo RS |  |  | |  | |
|  | Total | 14.4 ± 0.3 (13882) | 12.7 ± 0.3 (17506)*** | | 11.4 ± 0.7 (2228)** | |
|  | 0-34 | 25.0 ± 8.9 (24) | 18.2 ± 8.2 (22) | | 33.4 ± 27.2 (3) | |
|  | 35-49 | 19.9 ± 1.4 (783) | 17.2 ± 1.2 (957) | | 13.9 ± 2.7 (167) | |
|  | 50-64 | 19.1 ± 0.6 (4624) | 16.1 ± 0.5 (5854)** | | 15.1 ± 1.3 (821)* | |
|  | 65-79 | 12.1 ± 0.4 (7049) | 10.9 ± 0.3 (8956) | | 9.0 ± 0.9 (1053)* | |
|  | 80+ | 6.6 ± 0.7 (1402) | 7.2 ± 0.7 (1717) | | 5.8 ± 1.9 (184) | |
|  | 36-Mo RS |  |  | |  | |
|  | Total | 9.5 ± 0.3 | 8.2 ± 0.2** | | 6.8 ± 0.6** | |
|  | 0-34 | 16.7 ± 7.6 | 13.7 ± 7.3 | | 33.4 ± 27.2 | |
|  | 35-49 | 13.4 ± 1.2 | 12.0 ± 1.1 | | 6.7 ± 1.9 | |
|  | 50-64 | 12.7 ± 0.5 | 10.3 ± 0.4** | | 8.9 ± 1.0* | |
|  | 65-79 | 7.8 ± 0.3 | 7.1 ± 0.3 | | 5.5 ± 0.7* | |
|  | 80+ | 4.8 ± 0.7 | 4.5 ± 0.6 | | 4.1 ± 1.7 | |
| 03-12 | 24-Mo RS |  |  | |  | |
|  | Total | 15.4 ± 0.3 (17819) | 13.6 ± 0.2 (26739)*** | | 13.7 ± 0.5 (6045)* | |
|  | 0-34 | 25.0 ± 12.5 (12) | 26.8 ± 9.6 (25) | | 57.2 ± 18.7 (7) | |
|  | 35-49 | 22.6 ± 1.4 (905) | 19.0 ± 1.1 (1346) | | 19.8 ± 2.0 (401) | |
|  | 50-64 | 19.7 ± 0.5 (6226) | 16.4 ± 0.4 (9622)*** | | 16.3 ± 0.8 (2379)** | |
|  | 65-79 | 13.5 ± 0.4 (8442) | 12.4 ± 0.3 (12690) | | 11.8 ± 0.6 (2765) | |
|  | 80+ | 7.6 ± 0.6 (2234) | 6.6 ± 0.5 (3056) | | 6.1 ± 1.2 (493) | |
|  | 36-Mo RS |  |  | |  | |
|  | Total | 10.1 ± 0.2 | 8.9 ± 0.2*** | | 8.7 ± 0.4** | |
|  | 0-34 | 16.7 ± 10.8 | 21.5 ± 9.0 | | 57.2 ± 18.7 | |
|  | 35-49 | 15.7 ± 1.3 | 12.8 ± 0.9 | | 11.5 ± 1.7 | |
|  | 50-64 | 13.0 ± 0.5 | 10.9 ± 0.3** | | 10.3 ± 0.7* | |
|  | 65-79 | 8.7 ± 0.3 | 8.0 ± 0.3 | | 7.6 ± 0.6 | |
|  | 80+ | 4.7 ± 0.5 | 4.1 ± 0.4 | | 4.8 ± 1.2 | |

Abbreviations: Mo, month; RS, relative survival; SEM, standard error of the mean.

**p* < 0.01, ***p* < 0.001, and ****p* < 0.0001 for comparisons with the Low Poverty group.

**Supplementary Table S6.** Summary data for race distribution by SES and calendar period in patients with SCLC from 1983 to 2012 at 18 SEER sites.

| Decades | SES | Number | White | Black | Other |
| --- | --- | --- | --- | --- | --- |
| 1983-2012 |  |  |  |  |  |
|  | Total | 106295 | 87.59% | 8.40% | 4.02% |
|  | Low Poverty | 43729 | 93.23% | 3.57% | 3.20% |
|  | Medium Poverty | 53861 | 83.02% | 11.84% | 5.14% |
|  | High Poverty | 8705 | 87.56% | 11.30% | 1.14% |
| 1983-1992 |  |  |  |  |  |
|  | Total | 21027 | 88.71% | 7.85% | 3.44% |
|  | Low Poverty | 11530 | 95.99% | 2.97% | 1.03% |
|  | Medium Poverty | 9128 | 79.20% | 14.23% | 6.57% |
|  | High Poverty | 369 | 96.48% | 2.17% | 1.36% |
| 1993-2002 |  |  |  |  |  |
|  | Total | 33931 | 87.31% | 8.24% | 4.45% |
|  | Low Poverty | 14052 | 93.60% | 3.36% | 3.05% |
|  | Medium Poverty | 17643 | 82.26% | 11.76% | 5.98% |
|  | High Poverty | 2236 | 87.66% | 11.14% | 1.21% |
| 2003-2012 | |  |  |  |  |
|  | Total | 51337 | 87.32% | 8.72% | 3.96% |
|  | Low Poverty | 18147 | 91.18% | 4.11% | 4.71% |
|  | Medium Poverty | 27090 | 84.80% | 11.09% | 4.11% |
|  | High Poverty | 6100 | 86.98% | 11.92% | 1.10% |

**Supplementary Table S7.** Summary data for stage distribution by calendar period in patients with SCLC from 1983 to 2012 at 18 SEER sites.

|  | 1983-1992 | 1993-2002 | 2003-2012 |
| --- | --- | --- | --- |
| Localized | 7.00% | 6.00% | 5.00% |
| Regional | 22.00% | 24.00% | 21.00% |
| Distant | 62.00% | 64.00% | 71.00% |
| Unstaged | 10.00% | 5.00% | 3.00% |
